# The Prevailing Pandemic of Influenza

**Published:** 1918-11

**Authors:** 


**The Prevailing Pandemic of Influenza.** By J. J. Keegan, M. D. *The Journal of the American Medical Association*, September 28, 1918.

A severe and very rapidly spreading epidemic of influenza burst out in August, in the First Naval District. More than 2000 cases were reported within two weeks.

The disease is similar to the familiar endemic influenza, except that it is often more severe and extraordinarily contagious, and that serious complications are very frequent.

*Characteristics of the Disease.* The incubation period is only one to two days. The onset is very sudden. Fever rises rapidly from 101 to 105 F. (38.3 to 40.6 C.). Severe headache, weakness, general malaise, pains in the muscles and joints are the usual features. In uncomplicated cases remission occurs in the morning of the second or third day. Frequently there is a slight relapse of fever before the recovery. Blood cultures taken at all stages of the disease have proved negative — the disease seems to be a toxemia.

White blood cell count below normal. Differential leukocyte count essentially normal. The urine often shows faint traces of albumin.

About 5 to 10 per cent of the patients develop a very severe and massive bronchopneumonia, and among these mortality rises as high as 60 or 70 per cent.

The consolidation appears most frequently in the right inferior lobe, just medial to the inferior angle of the scapula. It extends very rapidly to the left inferior, the right upper, and middle lobes, and finally to the left superior lobe, so that in many cases death is due to asphyxiation.

In uncomplicated cases, leukopenia persists, since the influenza bacillus calls forth no defensive leukocytosis in the circulating blood. If secondary infection occurs, with either the pneumococcus or streptococcus, then a marked leukocytosis develops.

When bronchopneumonia occurs, there is frequently a serofibrinous effusion into the pleural cavity, most generally unilateral and on the right side, varying in amount from 100 to 1000 c. c.

Post-mortem examination of the lungs shows, in the prolonged cases, a characteristic condition of diffuse and confluent small abscesses. Creamy, grayish yellow pus exudes from the cut surface, leaving a slight reticulum of necrotic lung tissue.

The remaining organs are quite normal in appearance, but for a light degree of congestion.

*Bacteriologic Findings.* These have been very confused. No conclusive results seem to have been reported as yet as to the etiologic agent of this epidemic.

The influenza bacillus has been found only occasionally. Streptococci, pneumococci and the Micrococcus catarrhalis were recovered more frequently from the sputum and throat.

Researches made in the naval stations of Boston showed that the predominating organism in *throat cultures* was a gram-positive diplococcus, with a considerable green area, surrounding the small gray colonies. The researches for the influenza bacillus proved generally negative, either because of the difficulty of isolating this bacillus from mixed cultures, or because the focus of the influenza infection is not in the pharynx, but somewhere in some recess of the nasal cavity.

Smears cultures from the *lung punctures* or *necropsies*, gave more constant results. The influenza bacillus occurred in 82.6 per cent of the cases. The pneumococcus appeared as secondary invader in 56.5 per cent of these cases. Type 1 pneumococcus was never met with. The hemolytic Streptococcus occured in 22.7 per cent of these cases. It must be noted that this percentage is very low compared to the incidence of this streptococcus in the postmeasles bronchopneumonias of the past winter.

*The Influenza Bacillus.* According to these findings the influenza bacillus seems to be the primary invader in all cases of influenza complicated by pneumonia. This is always a bronchopneumonia frequently associated with a secondary infection with the pneumococcus, or the streptococcus, and less frequently with the Micrococcus catarrhalis and the Staphylococcus aureus. These multiple infections may be due to a well-recognized property of the influenza bacillus to grow much more luxuriantly, and acquire a much larger size in the presence of certain organisms : staphylococcus, pneumococcus and streptococcus.

Morphologically the influenza bacilli are exceedingly small gram-negative coccobacilli, grouped in colonies of very small size and colorless appearance. No specific agglutination or fermentation tests are available as yet for further identification of this organism.

An experiment has been performed on nine volunteers who had never been exposed to the disease, in order to determine whether or not influenza was due to a filtrable virus. Filtrates from nasal and throat washings from two typical cases were introduced into the anterior nares of these volunteers. None of these showed symptoms of influenza during ten days of isolation.

